# Exercise unveiled: an integrated model of motives, goals, affective states, and stress during the COVID-19 pandemic

**DOI:** 10.1186/s40359-026-04693-1

**Published:** 2026-05-09

**Authors:** Asli Elif Aydin

**Affiliations:** Faculty of Business, Istanbul Bilgi University, Istanbul, Türkiye

**Keywords:** Exercise behavior, Exercise participation motives, Exercise regulatory motives, Pandemic

## Abstract

**Background:**

This study develops and tests an extended model to examine exercise behavior during the COVID-19 pandemic. In addition to exercise participation goals and regulatory styles, the model incorporates affective states and stress-related constructs, which are particularly relevant in the context of the pandemic.

**Methods:**

Data were collected from a sample of 283 participants during the pandemic, a period marked by heightened stress and disrupted routines. Structural equation modeling was employed to test the proposed relationships among exercise participation goals, behavioral regulation, affective states, stress, and exercise behavior.

**Results:**

The results reveal that physical and psychological goals significantly predict exercise behavior. Regarding behavioral regulation, intrinsic and introjected regulation are positively associated with exercise behavior. Furthermore, negative affect and the ability to cope with stress show significant relationships with exercise behavior. The findings also indicate significant mediating effects of amotivation, introjected regulation, and intrinsic regulation on the relationship between participation goals and exercise behavior. Additionally, exercise participation goals mediate the effects of positive and negative affect, as well as the ability to cope with stress, on exercise behavior.

**Conclusions:**

These findings highlight the interplay of motivational, affective, and stress-related factors in shaping exercise behavior during the pandemic. The study provides practical implications for designing exercise promotion and intervention programs, particularly in contexts of public health crises.

**Supplementary Information:**

The online version contains supplementary material available at 10.1186/s40359-026-04693-1.

## Background

Exercise is essential for both physical and mental well-being [1, 2]. Lack of exercise may result in physiological problems, such as weight gain, increased blood pressure, and insulin resistance [3], while regular exercise may help prevent mental problems like depression, anxiety, and insomnia [4, 5]. Despite these benefits, sedentary lifestyles have become increasingly prevalent due to a lack of time, limited access to exercise facilities, and the rise of passive leisure activities such as video gaming and social media use [6]. The COVID-19 pandemic exacerbated this trend, significantly declining physical activity levels during and even after lockdowns and social distancing measures [7]. Understanding the factors influencing exercise behavior during such periods of disrupted routines, heightened stress, and altered affective states is therefore particularly important.

The present study proposes an extended model that examines adherence to exercise through the lens of self-determination theory (SDT) [8], while incorporating perceived stress, affective states, stress-coping ability, participation goals, and behavioral regulation. Although prior research has examined some of these constructs in isolation, few studies have integrated them within a single framework or examined their interplay in a crisis context. By addressing this gap, the current study seeks to clarify how affective and stress-related processes shape exercise goals, motivational regulation, and ultimately exercise behavior during a period characterized by uncertainty and disruption.

### Conceptual framework

#### Behavioral regulation

SDT posits that motivation can be understood along a continuum of behavioral regulation ranging from fully self-determined (autonomous motivation**)** to non-self-determined (controlled motivation**)** [8, 9]. Behavioral regulation captures qualitative differences in how exercise behavior is motivated. Within this continuum, five primary forms of regulation increase in self-determination: amotivation, external regulation, introjected regulation, identified regulation, and intrinsic regulation [10].

Intrinsic regulation reflects the most autonomous form of regulation, driven primarily by the fun and enjoyment of the exercise act [11]. A state of decreased autonomy is identified regulation, in which exercise behavior is motivated by individuals' values regarding exercise [10]. A partially controlled type of regulation is introjected regulation, which involves engaging in exercise to reduce guilt and embarrassment or to improve one's self-image [12]. External regulation, a form of controlled motivation, occurs when exercise behavior is motivated by external rewards and punishments [11]. Lastly, amotivation reflects the absence of intentional regulation, often occurring when individuals perceive little connection between their actions and outcomes [10].

Evidence from prior work indicates that, while more autonomous forms of regulation, particularly intrinsic and identified regulation, predict adherence to exercise for the long term, introjected regulation predicts shorter-term engagement [13, 14]. In contrast, external regulation is shown to have either a neutral or a negative effect on commitment to exercise participation, while amotivation predicts low intention and disengagement from exercise behavior [11]. Accordingly, it is hypothesized that:

- H1: Intrinsic, identified, and introjected regulation will be positively associated with exercise behavior, whereas external regulation and amotivation will be negatively associated with exercise behavior.

### Exercise-participation goals

Exercise-participation goals, which reflect the expected outcomes of exercise behavior [15], also play a key role in shaping exercise behavior [11, 16]. Individuals might exercise to attain physical goals (e.g., fitness, health, weight, appearance) and psychological goals (e.g., fun, stress relief, mental health) [17, 18]. Individuals may hold multiple goals simultaneously.

Earlier studies report significant associations among exercise participation goals, regulatory behavior, and adherence to exercise; for instance, one study reveals that high-attendant members of a fitness center are distinguishable from less-attendant ones based on fun, mastery, and social goals [16]. It is also indicated that physical goals related to weight and appearance, which are associated with external regulation, are more influential during the initiation phase of exercise, whereas recreational goals become more prominent in maintaining exercise behavior [11]. Overall, exercise participation goals are expected to predict exercise behavior, such that:

- H2: Both physical and psychological exercise goals will be positively associated with exercise behavior.

In addition, exercise participation goals are expected to influence how individuals regulate their exercise behavior. Within the self-determination theory framework, the internalization of goals plays a central role in determining the type of behavioral regulation individuals adopt. Stronger, internalized goals emphasizing physical and psychological benefits are more likely to foster autonomous and internally driven forms of regulation, while weakly internalized goals may be associated with externally driven forms of regulation and amotivation [16]. For instance, it is indicated that stronger goals, aligned with personal interests, promote higher levels of intrinsic motivation [19]. While one study found a positive relationship between physical goals and introjected regulation [20], others reported that when exercise goals lack strength or personal significance, individuals may rely on controlled motivations that do not reinforce enduring behavioral change or amotivation [21, 22]. Therefore, it is hypothesized that:

- H3 & H4: Physical (H3) and psychological (H4) exercise participation goals will be positively associated with intrinsic, identified, introjected regulation and negatively associated with external regulation and amotivation.

### Affect and stress

Affective states (i.e., individuals’ momentary or general emotional experiences, including positive and negative feelings) further contribute to variations in exercise behavior. Prior studies established significant associations between affect and physical activity, revealing bidirectional relationships. It is widely acknowledged that exercise impacts emotion and mood, leading to reduced negative affect and heightened positive affect [23, 24]. It is demonstrated that increased negative affect is linked to reduced physical activity levels [25], and a study conducted during the pandemic reports that individuals with a negative emotional state have a diminished intention to exercise [26].

In the opposite direction, it is also reported that individuals with a negative mood might engage in exercise to overcome their affective state to self-regulate [27]. Still, most studies report that individuals are more likely to remain physically inactive when the core emotional valence associated with inactivity is more positive than that of exercise [28]. Meanwhile, positive affect is also associated with increased exercise through an affect-maintenance mechanism such that individuals engage in exercise behavior to preserve their positive state and well-being [29]. Moreover, it is suggested that participants were more inclined to exercise when they had experienced higher levels of positive incidental affect earlier in the day [30].

Similarly, stress-related constructs such as perceived stress and coping with stress also influence exercise behavior. Affect and stress are related but distinct constructs. Perceived stress reflects cognitive appraisal of demands [31], while coping reflects individuals’ capacity to manage these demands [32]. Affect, in turn, captures the emotional experiences accompanying such appraisals. Higher perceived stress is typically linked to increased negative and reduced positive affect [33], whereas greater coping ability is linked to more positive and less negative affect [34]. Despite these associations, the constructs represent different processes and are modeled separately.

As with affect, stress shows reciprocal associations with exercise motives and behavior. Prior research suggests that regular exercise can reduce perceived stress and enhance individuals’ capacity to cope with it [35]. In the opposite direction, higher stress levels have been linked to lower exercise participation [36], and individuals who report lower stress tend to engage in physical activity more frequently [37]. One explanation for this pattern is that stressed individuals may feel pressed for time or emotionally depleted, leading them to deprioritize exercise in the face of competing demands [24].

Although prior research documents reciprocal relationships between affect, stress, and exercise behavior, the present study conceptualizes affect and stress as antecedent conditions shaping exercise participation goals and behavior. This focus is particularly appropriate in the context of the pandemic, when emotional responses and heightened stress were largely driven by contextual disruptions rather than by exercise itself. The model positions affect and stress as upstream psychological states influencing goal formation and motivational processes prior to behavioral engagement. Given the cross-sectional design, this specification provides a parsimonious and theoretically grounded approach to examining exercise behavior under crisis conditions. Accordingly, it is hypothesized that:

- H5: Positive affect will be positively associated with exercise behavior, while negative affect will be negatively associated with exercise behavior.
- H6: Perceived stress will be negatively associated with exercise behavior, while stress-coping ability will be positively associated with it.

Moreover, affective states are expected to influence exercise goals that individuals set for themselves. Specifically, it is indicated that people in a negative affective state experience a reduced perceived self-efficacy and increased focus on immediate relief, hindering their ability to set future-oriented goals [38]. Likewise, individuals experiencing negative affect due to pandemic conditions, such as isolation or uncertainty, may be less likely to set fitness or health-related goals, potentially reducing their exercise adherence. Conversely, it is suggested that people in a positive affective state are more inclined to set goals and believe they can accomplish them [39]. Positive affect, therefore, may enhance goal-setting and motivation, promoting sustained physical activity.

Similar to affect, stress-related constructs are also expected to influence exercise-related goals. While individuals with greater stress-coping ability may be better able to maintain exercise behavior despite adverse conditions, those with higher perceived stress may struggle to exercise. Prior research reports that individuals who are better at managing their stress through methods such as self-compassion and mindfulness show increased motivation to exercise [40]. Moreover, a positive relationship is found between being less overwhelmed with stress and the pursuit of goals [41]. Accordingly, it is hypothesized that:

- H7: Positive affect will be positively associated with physical and psychological exercise goals, while negative affect will be negatively associated with these goals.
- H8: Perceived stress will be negatively associated with physical and psychological exercise goals, while stress-coping ability will be positively associated with these goals.

Apart from the direct effects of exercise participation goals, affect, and stress on exercise behavior, indirect effects are also posited. Prior studies indicate that goals influence motivational regulation, which, in turn, predicts exercise behavior, suggesting a sequential pathway from goal endorsement to regulatory style to behavior [11, 42]. Based on that;

- H9: Behavioral regulations (intrinsic/introjected/identified/external regulation/amotivation) will mediate the relationship between exercise participation goals (physical/psychological) and exercise behavior.

Similarly, appraisal-based perspectives suggest that affective states and stress influence exercise behavior indirectly by shaping individuals’ goal priorities rather than exerting only direct effects. Stress can undermine motivation and goal setting by increasing feelings of being overwhelmed, thereby limiting individuals’ capacity to focus on and pursue exercise-related goals [43]. In contrast, effective coping may support sustained engagement by enabling individuals to manage these demands. Affective states may likewise facilitate or hinder motivation to pursue exercise participation goals, as individuals in more positive emotional states are more likely to initiate and maintain goal-directed behavior, whereas negative affect may reduce such motivation [44]. Accordingly, exercise participation goals are expected to transmit the effects of affect and stress-related variables on exercise behavior. Accordingly;

- H10: Exercise participation goals (physical/psychological) will mediate the relationship between affect/stress variables and exercise behavior.

**Fig. 1:**
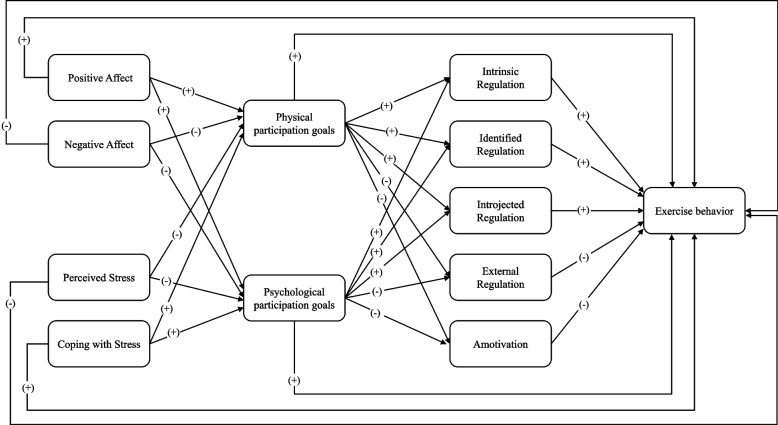
The research model

Drawing on these theoretical foundations, this study develops a comprehensive model integrating SDT-based regulatory processes, exercise participation goals, stress and coping constructs, and affective states to explain exercise behavior during the pandemic. The proposed model (Fig. 1) positions affective states and stress as antecedents to both exercise goals and behavioral regulation, which, in turn, influence exercise behavior. This structure suggests that affective and stress responses shape cognitive and motivational processes before influencing behavioral outcomes. By situating exercise behavior within this broader set of psychological influences, the model provides a framework for understanding how individuals navigated exercise-related decisions during the unprecedented conditions of the COVID-19 pandemic.

## Methods

### Data

The study data were collected in Turkey in 2021, during the COVID-19 pandemic. During data collection, government measures existed to "flatten the curve" (i.e., to prevent peak infection rates) such as social distancing, dusk-to-dawn curfews, and weekend quarantines. An online survey was conducted with a convenience sample due to the difficulty of collecting data under pandemic restrictions. Undergraduate and graduate students at a university in İstanbul, Turkey, were recruited for the study. Students were invited to the study through LMS posts of several courses and email announcements. 436 students were invited to participate in the study via email with links to the online survey in exchange for course credit. Instead of participating, they were also given the option to complete a short assignment. Initially, 291 students responded. However, eight were eliminated for failing to respond to an attention-testing question that required a standard response (e.g., “please select strongly agree for this item”).

The final sample consisted of 283 participants. The sample mean age was 26 years (standard deviation [SD] = 8.8); 53.4% of the participants were female, and their average body mass index was 22.6 kg/m^2^ (SD = 3.4). The average household size of the participants was 3.3 (SD = 1.7) people. The percentages of participants who engaged in minimal, moderate, and strenuous activity at least 3 times per week were 43.3%, 38.1%, and 35%, respectively. 26.5% reported not engaging in any activities to work up a sweat during the pandemic (Table 1).

**Table 1:** Sample characteristics

**Variable**	**Category**	**Freq**	**%**		**Variable**	**Category**	**Freq**	**%**
Sex	Male	132	46.6		Strenuous activity (times per week)	0	86	30.4
	Female	151	53.4			1–2	98	34.6
						3–4	69	24.4
Age (years)	18–20	132	46.6			≥ 5	30	10.6
	21–30	72	25.4					
	31–40	50	17.7		Moderate activity (times per week)	0	56	19.8
	≥ 41	29	10.4			1–2	119	42
						3–4	70	24.7
BMI	< 18.5	23	8.1			≥ 5	38	13.4
	18.5–24.9	197	69.6					
	25–29.9	50	17.7		Minimal activity (times per week)	0	60	21.2
	≥ 30	13	4.6			1–2	101	35.7
						3–4	67	23.7
Household size	1	36	12.7			≥ 5	55	19.6
	2	45	15.9					
	3–4	160	56.5		Sweat	Often	84	29.7
	≥ 5	42	15			Sometimes	124	43.8
						Never/rarely	75	26.5

Data were collected online using Google Forms. Before starting the survey, participants were asked to provide informed consent. Responses were recorded anonymously. Upon completion, participants were directed to a confirmation page to grant course credit. The questionnaire was administered in Turkish, having been translated from English using a standard translation and back-translation procedure.

### Measures

The survey included scales assessing participants' exercise behavior, exercise-participation goals, and their motives to exercise. Moreover, items assessing participants' positive and negative affect, coping ability, stress level, extent of confinement, and demographic characteristics were also included. The majority of the constructs were measured using established and widely validated scales from prior research, including the Behavioral Regulation in Exercise Questionnaire-2 (BREQ-2) [10], the Leisure-Time Exercise Questionnaire [42], affect measures based on prior work [43], and stress-related single-item measures validated by Littman et al. [44]. Exercise participation goals were adapted from Rogers et al. [18]. Minor adaptations were made during translation to improve clarity and cultural appropriateness; for example, some items are slightly rephrased to improve linguistic clarity. Response formats varied across scales because each instrument was administered using its originally validated Likert format in order to preserve reliability and comparability with prior studies.

In the current study, the reliability and validity of multi-item constructs were assessed through confirmatory factor analysis, composite reliability (CR), and average variance extracted (AVE), with all constructs meeting recommended thresholds (see Table 3).

#### Exercise behavior

The leisure-time exercise questionnaire [42] was employed to measure participants' exercise behavior. Participants were asked to answer, "In the last month, in a typical 7-day period, how many times on average did you do the following kinds of exercise for more than 15 min during your free time?" Participants reported their weekly frequency of exercise in three different categories: mild (e.g., yoga, easy walking), moderate (e.g., fast walking, easy bicycling), and strenuous (e.g., running, basketball) exercise. A total leisure activity score was computed by summing the products of weekly frequencies of mild, moderate, and strenuous exercise with 3, 5, and 9 points, respectively. In addition, "the frequency of weekly leisure-time activities pursued long enough to work up a sweat" was measured on a scale ranging from (1 point) "never/rarely" to (3 points) "often."

#### Exercise-participation goals

Participants' reasons for exercising were examined using two measures assessing psychological (e.g., feeling less bored) and physical goals (e.g., building muscle strength). Four items adapted from Rogers et al.'s study were used for each scale [18]. Participants indicated how often each participation goal gets them to exercise. Responses ranged from (1 point) "never" to (5 points) "very often."

##### Behavioral regulation in exercise

Exercise motivations were assessed using the Behavioral Regulation in Exercise Questionnaire-2 [10]. This questionnaire includes 19 items covering five dimensions representing various degrees of self-determination: amotivation (e.g., I do not see the point in exercising), external regulation (e.g., Because other people say I should), introjected regulation (e.g., Because I feel guilty when I do not exercise), identified regulation (e.g., Because I value the benefits of exercise), and intrinsic motivation (e.g., Because I enjoy my exercise sessions). A 5-point Likert scale was employed, with responses ranging from (0 points) "not true for me" to (4 points) "very true for me."

#### Affect

Measures of both positive and negative affects [43] were included in the questionnaire. Participants were asked to indicate how much they experienced each of the six feelings last month (e.g., Joyful, content, interested, angry, anxious, low-spirited). A 7-point Likert scale was employed, with responses ranging from (0 points) "not at all" to (6 points) "very much."

#### Stress

Two single-item measures [44] were used to assess participants' perceived stress and coping ability. It was found that the test–retest reliabilities of the measures were good, and the validity of both measures was comparable to that of stress measures with more items [44]. Participants were first asked to report their ability to cope with stress last month on a 6-point scale ranging from (1) "I can shake off stress" to (6) "stress eats away at me." Next, participants were asked to rate the level of stress in their lives over the last month on a 6-point scale ranging from (1) "no stress" to (6) "extreme stress."

#### Confinement

The extent of confinement that most individuals experienced during the pandemic may also have influenced their exercise motives and behavior. Since the level of confinement varied significantly among individuals during and after the pandemic, the extent of confinement experienced was also included in the model to control for its impact. Confinement was measured using a single-item scale capturing the extent to which individuals restricted their movement during the pandemic. Participants responded on a 7-point scale ranging from 1 = “I left home as often as before the pandemic” to 7 = “I stayed at home all the time,” with higher values indicating greater levels of confinement. The scale was designed to reflect a continuum of perceived behavioral restriction rather than mutually exclusive categories, allowing participants to indicate relative changes in their mobility compared to pre-pandemic conditions.

#### Demographic characteristics

Participants were asked to indicate their weight, height, age, and sex at the end of the questionnaire. Then, based on weight and height, the body mass index was computed. Sex was coded as 1 (female), and age was measured as a continuous variable in years.

### Data analysis

Prior to analysis, data were screened for missing values and for violations of distributional assumptions. The amount of missing data was minimal (less than 3%). Missing values were handled using mean imputation at the scale level, which is considered appropriate when the proportion of missing data is low. Assumptions of normality were evaluated by examining skewness and kurtosis values for all study variables, which fell within acceptable ranges.

Structural Equation Modeling (SEM) was used to examine the relationship between the study variables. Data entry and descriptive analysis were performed using IBM SPSS Statistics 22, and SEM analysis was performed using IBM Amos 22. The study adopted a significance level of 0.05.

The model's fit was evaluated using several fit indices, such as the Ratio of chi-square minimum to degrees of freedom (CMIN/df), Comparative Fit Index (CFI), Tucker-Lewis Index (TLI), Incremental Fit Index (IFI), and Root Mean Square Error of Approximation (RMSEA). Detailed findings for these fit indices are presented in the results section.

The mediating effects were tested using Preacher and Hayes' [45] bootstrapping procedure in Amos 22.0 as well. A bootstrap analysis with 2000 resamples was performed using 95% bias-corrected, percentile-based confidence intervals.

## Results

### Measurement model

The measurement model was tested before testing the research model depicted in Fig. 1. Prior studies using the Behavioral Regulation Scale with Turkish participants employed only 4 of its dimensions [46, 47]. The Turkish version of the scale, which does not include the identified regulation dimension, showed high reliability and validity. Still, an exploratory factor analysis using maximum likelihood estimation with Promax rotation was performed to test the dimensionality of the full version of the scale. The inspection of the factor analysis results for factor loadings, communalities, and eigenvalues indicated that four factors exist. The items of identified regulation cross-load on the intrinsic regulation factor, and the loadings were below the recommended threshold of 0.4 [48]. Therefore, identified regulation items were removed from the analysis. Moreover, two items of external motivation, one item of amotivation, and one item of psychological goals were eliminated due to low factor loadings. The descriptive statistics and Pearson correlations of the final constructs are demonstrated in Table 2. Correlations among the constructs were within acceptable ranges, and variance inflation factor (VIF) values were below commonly accepted thresholds, indicating no evidence of problematic multicollinearity.

**Table 2:** Descriptives and correlations between constructs

Constructs	A	B	C	D	E	F	G	H	I	J	K	L
A. Exercise behavior	1											
B. Physical goals	0,43**	1										
C. Psychological goals	0,37**	0,57**	1									
D. Ext. Regulation	−0.13	−0.13	−0,03	1								
E. Intrj. Regulation	0,27**	0,34**	0,31**	−0.12	1							
F. Int. Regulation	0,37**	0,37**	0,29**	−0,24**	0,46**	1						
G. Amotivation	−0,28**	−0,22**	−0,19*	0,35**	−0,34**	−0,33**	1					
H. Positive affect	0,19*	0,26**	0.13	0.08	0.04	0.07	0.04	1				
I.Negative affect	−0,10	0,16*	0,28**	−0.08	0.10	0.12	−0.01	−0,35**	1			
J. Stress coping ability	0.18	0.04	0,21*	0.17	0.13	−0,21*	0.00	−0,33**	0,41**	1		
K. Stress level	−0,08	0,24**	0,29**	−0,16	0,13	0,08	−0,01	−0,39**	0,83**	0,46**	1	
L. Confinement	−0,3**	0,06	0,02	0,19	−0,56	−0,03	−0,01	−0,56	0,19**	0,08	0,2**	1
Mean	19.75	3.75	3.17	0.53	1.76	2.52	0.25	3.10	3.43	3.22	3.84	5.30
Std. Deviation	13.76	1.04	1.17	0.75	0.98	1.12	0.48	1.33	1.54	1.41	1.42	1.52

^*^p<0.05

^**^p<0.01

Next, a confirmatory factor analysis was performed to evaluate the reliability and validity of the scales. Latent constructs with multiple indicators were analyzed using maximum likelihood estimation in Amos 22.0 (IBM Corporation). Fit indices indicated that the measurement model provides a good fit ([CMIN/df] = 1.616, IFI = 0.96, TLI = 0.95, CFI = 0.96, RMSEA = 0.047). Convergent validity of the constructs was assessed using factor loadings, average variance extracted (AVE), and composite reliability. All items loaded significantly (*p <* 0.01) onto their constructs, with factor loadings ranging from 0.60 to 0.94 (Table 3). All AVE scores exceeded the required threshold of 0.5 except for the introjected motivation construct. Still, convergent validity was established, as the composite reliability for this construct exceeded 0.6 [49]. Discriminant validity was checked by comparing the square root of the AVE score of a construct against the correlation coefficients between the construct and the model's remaining constructs [49]. AVE scores were higher for all constructs, providing evidence for discriminant validity.

**Table 3:** Confirmatory factor analysis results

Constructs/items	Factor loading	CR	AVE
*Exercise behavior*		0.70	0.54
Weekly frequencies of strenuous, moderate, and light activities	0.76		
Weekly leisure-time activities pursued long enough to work up a sweat	0.70		
*Physical goals*		0.91	0.71
Building muscle strength	0.77		
Increased physical fitness	0.93		
Increased energy	0.91		
Improving health	0.75		
*Psychological goals*		0.90	0.76
Feeling less bored	0.83		
Feeling less stressed	0.93		
Less depression	0.85		
*Intrinsic regulation*		0.93	0.77
Because I think exercise is fun	0.75		
Because I enjoy my exercise sessions	0.92		
Because I find exercise a pleasurable activity	0.93		
Because I get pleasure and satisfaction from participating in exercise	0.89		
*Introjected regulation*		0.73	0.48
Because I feel guilty when I don’t exercise	0.71		
Because I feel ashamed when I miss an exercise session	0.60		
Because I feel like a failure when I haven’t exercised in a while	0.76		
*External regulation*		0.79	0.66
Because other people say I should	0.66		
Because my friends/family/partner say I should	0.94		
*Amotivation*		0.77	0.53
I can’t see why I should bother exercising	0.69		
I don’t see the point in exercising	0.69		
I think exercising is a waste of time	0.81		
*Positive affect*		0.89	0.72
Joyful	0.84		
Content	0.94		
Interested	0.77		
*Negative affect*		0.81	0.59
Angry	0.70		
Anxious	0.73		
Low-spirited	0.88		

### Structural model

After confirmatory factor analysis, the structural model was tested using maximum likelihood estimation in Amos 22.0. The overall fit of the model was good ([CMIN/df] = 1.74, IFI = 0.98, TLI = 0.93, CFI = 0.97, RMSEA = 0.05). First, there was a significant positive relationship between psychological goals and introjected regulation. Physical goals showed significant associations with all four types of behavioral regulation; notably, the relationships were negative with external motivation and amotivation but positive with intrinsic and introjected motivation.

Results demonstrated that positive and negative affect constructs were significantly related to physical and psychological goals. An increase in both positive and negative affective states led to increased physical and psychological participation goals. Moreover, a significant relationship was observed between stress and physical goals. Conversely, the relationship between stress and psychological goals was not significant (Table 4).

**Table 4:** Path Estimates

Path	Standardized path coefficient	*p*
External regulation → Exercise behavior	−0,03	0,51
Intrinsic regulation → Exercise behavior	0,16	**
Introjected regulation → Exercise behavior	0,15	**
Amotivation → Exercise behavior	−0,09	0,07
Physical goals → Exercise behavior	0,27	***
Psychological goals → Exercise behavior	0,20	***
Physical goals → External regulation	−0,19	*
Physical goals → Intrinsic regulation	0,32	***
Physical goals → Introjected regulation	0,27	***
Physical goals → Amotivation	−0,18	*
Psychological goals → External regulation	0,08	0,271
Psychological goals → Intrinsic regulation	0,12	0,095
Psychological goals → Introjected regulation	0,21	**
Psychological goals → Amotivation	−0,11	0,134
Positive affect → Exercise behavior	0,07	0,16
Negative affect → Exercise behavior	−0,24	***
Perceived stress → Exercise behavior	−0,01	0,81
Stress coping → Exercise behavior	0,13	**
Positive affect → Physical goals	0,42	***
Positive affect → Psychological goals	0,33	***
Negative affect → Physical goals	0,24	***
Negative affect → Psychological goals	0,36	***
Perceived stress → Physical goals	0,17	*
Perceived stress → Psychological goals	0,12	0,09
Stress coping → Physical goals	−0,01	0,86
Stress coping → Psychological goals	0,08	0,19
Confinement → Exercise behavior	−0,17	***

^*^p < 0.05

^**^p < 0.01

^***^p < 0.001

Both intrinsic and introjected regulation had significant, positive relationships with exercise behavior, providing partial support for H1. The negative association between amotivation and exercise behavior was only marginally significant and therefore did not provide additional support for H1. External regulation, meanwhile, was not significantly related to exercise behavior.

The analyses further indicated a significant positive relationship between physical and psychological goals and exercise behavior, providing support for H2.

There were also significant paths from physical and psychological goals to introjected regulation and from physical goals to intrinsic, external regulation, and amotivation, providing partial support for H3 and H4.

While negative affect had a significant negative relationship with exercise behavior, the association between positive affect and exercise behavior was not significant, providing partial support for H5. The association between perceived stress and exercise behavior was not significant; however, a significant positive association between the ability to handle stress and exercise behavior was observed, providing partial support for H6.

**Fig. 2:**
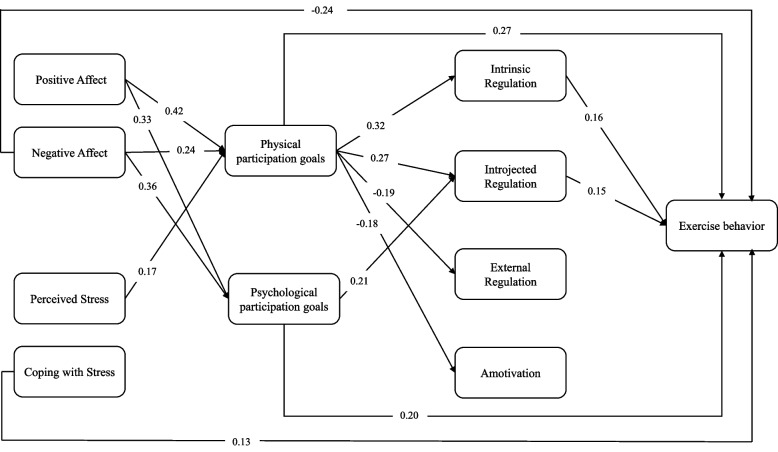
Structural Model with Significant Paths

The relationships between positive affect and both physical and psychological exercise participation goals were significant; however, contrary to expectations, negative affect was also positively associated with both types of goals, rather than negatively as hypothesized in H7. The relationship between perceived stress and physical goals was significant and positive. In contrast, its association with psychological goals was only marginally significant, providing partial support for H8 but in the opposite direction of the hypothesized effects. Lastly, there was a significant negative relationship between the extent of confinement and exercise behavior. All other paths were not significant. Figure 2 presents a simplified version of the structural model including only statistically significant paths.

### Mediation analyses

First, indirect effects were examined using behavioral regulation types as mediators (H9). The results demonstrated that introjected regulation mediates the effects of both physical and psychological goals on exercise behavior, whereas intrinsic regulation mediates the effects of physical goals on exercise behavior (Table 5a).

**Table 5:** Mediation effects

Total indirect effects	Bootstrap estimates		95% CI	
	**β**	*P*	**Lower**	**Upper**
(a) Behavior regulation types as mediators				
Physical goals → Ext. regulation → Exercise behavior	0.10	0.44	− 0.21	0.57
Psychological goals → Ext. regulation → Exercise behavior	− 0.04	0.34	− 0.33	0.06
Physical goals → Intr. Regulation → Exercise behavior	0.93	**	0.29	1.95
Psychological goals → Intr. Regulation → Exercise behavior	0.30	0.06	− 0.01	0.90
Physical goals → Intrj. Regulation → Exercise behavior	0.72	**	0.19	1.56
Psychological goals → Intrj. Regulation → Exercise behavior	0.51	**	0.14	1.10
Physical goals → Amotivation → Exercise behavior	0.29	*	0.01	0.90
Psychological goals → Amotivation → Exercise behavior	0.16	0.10	− 0.03	0.63
(b) Exercise participation goals as mediators				
Positive affect → Psychological goals → Exercise behavior	0.96	***	0.42	1.71
Negative affect → Psychological goals → Exercise behavior	1.05	***	0.45	1.94
Stress level → Psychological goals → Exercise behavior	0.27	0.06	− 0.01	0.76
Stress coping → Psychological goals → Exercise behavior	0.18	0.15	− 0.07	0.58
Positive affect → Physical goals → Exercise behavior	1.64	***	0.85	2.70
Negative affect → Physical goals → Exercise behavior	0.94	***	0.39	1.71
Stress level → Physical goals → Exercise behavior	0.53	*	0.11	1.14
Stress coping → Physical goals → Exercise behavior	− 0.03	0.90	− 0.41	0.36

^*^P < 0.05

^**^P < 0.01

^***^P < 0.001

The second set of analyses was conducted using exercise-participation goals as mediators (H10). It was shown that psychological goals mediate the effects of both positive and negative affect on exercise behavior. Regarding the mediating role of physical goals, significant effects existed for both positive and negative affect on exercise behavior. Lastly, perceived stress was indirectly associated with exercise behavior, with physical goals mediating this effect (Table 5b).

Overall, significant indirect effects through behavioral regulation and exercise participation goals provided partial support for H9 and H10.

## Discussion

The study results demonstrated that increases in intrinsic and introjected regulation also increase exercise behavior (providing partial support for H1). When individuals' reasons for exercising involve enjoying themselves and avoiding shame, they exercise more. Earlier studies also demonstrate the positive relationship between intrinsic regulation, introjected regulation, and exercise participation [12, 17]. In contrast, external regulation and amotivation were not significantly associated with exercise behavior. While prior research often reports negative or weak associations for these less self-determined forms of regulation, such effects are not always consistent [11, 50].

Regarding the goal-behavior relationship, it is shown that participation goals for physical and psychological exercise predict exercise behavior (supporting H2). This finding is consistent with prior research [11, 16, 51]. Individuals who expect to attain physical goals such as increased energy and psychological goals such as reduced stress tend to engage in more exercise. It is likely that clearly defined goals may strengthen behavioral engagement by providing direction and personal relevance to exercise.

The next set of findings relates to the relationship between exercise participation goals and behavioral regulation. Physical participation goals were significantly associated with all dimensions of behavioral regulation (supporting H3). Notably, increased physical goals were positively associated with intrinsic and introjected regulation but negatively associated with external regulation and amotivation. This pattern suggests that stronger endorsement of physical goals may facilitate the internalization of exercise behavior, shifting motivation toward more self-determined forms while reducing reliance on externally controlled motives. In other words, individuals who set clear goals such as increasing their stamina and improving their health are more likely to engage in exercise for enjoyment or personal standards rather than external pressures. These findings are consistent with prior research [19–22].

Besides, a positive relationship was revealed between psychological goals and introjected regulation (partially supporting H4). In other words, those motivated to feel less bored and less depressed by exercise are more likely to be driven by a desire to avoid guilt. This finding is inconsistent with earlier studies' findings, which mainly demonstrated that goals such as mental and physical health are associated with intrinsic regulation [17, 52]. One possible explanation is that psychological goals in this study primarily reflect the desire to reduce negative states such as boredom, stress, and depression rather than to enhance enjoyment. As a result, exercise may be driven by internal pressure to feel better, aligning more closely with introjected regulation than intrinsic motivation.

Next, it was shown that, while negative affect have a negative impact, the ability to cope with stress positively influences exercise behavior (providing partial support for H5 and H6). As the level of confinement, apprehension and worry increases, people exercise less. However, as the ability to handle stress increases, so too does participation in exercising. This pattern suggests that negative emotional states may act as psychological barriers to action, whereas coping ability may facilitate behavioral engagement by enabling individuals to manage stress more effectively. In contrast, positive affect and perceived stress were not significantly associated with exercise behavior, suggesting that their effects may operate indirectly through motivational processes rather than exerting direct influence. These findings also corroborate prior results [25, 26, 37].

The study further revealed that positive associations exist between affect and exercise-participation goals; specifically, those experiencing positive affect have enhanced physical and psychological goals, providing partial support for H7. As in prior studies, positive affective states, such as contentment and joy, likely heighten individuals' expectations of exercise outcomes [53].

Since people in a positive affective state are more willing to establish goals and believe they can attain them, positive affect, even in the context of a pandemic, may enhance goal-setting and motivation for both physical and psychological goals. Regarding the relationship between negative affect and exercise goals, however, the observed link was opposite to that hypothesized in H7. Contrary to expectations, higher levels of negative affect were also associated with stronger physical and psychological exercise goals. One likely explanation for this finding is that individuals may be motivated to overcome or regulate negative affective states, such as anxiety or low mood, thereby strengthening the salience of exercise-related goals as a coping mechanism [27].

Another finding relates to stress-related variables. The ability to cope with stress was not significantly associated with participation goals but was positively associated with exercise behavior. This pattern suggests that coping may facilitate the translation of motivation into action rather than shaping goal content. In other words, individuals with higher coping ability may engage in exercise as a direct strategy for managing stress, rather than as a means of achieving explicitly defined outcomes. Thus, exercise may function as a self-regulation or coping behavior rather than a goal-driven activity, consistent with prior research [54]. This also implies that individuals with stronger coping capacity may rely less on explicit goal-setting, engaging in exercise through more habitual or automatic responses to stress.

Perceived stress, on the other hand, is significantly associated with participation goals. Higher levels of perceived stress are associated with stronger physical exercise participation goals. A similar pattern was also observed for psychological goals, though only at the marginal significance level. These findings provide partial support for H8, but in the opposite direction of the hypothesized effects. Similar to the effect observed for negative affect, heightened stress may increase the perceived utility of exercise as a means of regulation, thereby strengthening individuals’ exercise-related goals. Given widespread awareness of the stress-reducing benefits of exercise, this pattern is plausible and aligns with prior research [41].

Moreover, the results indicated significant mediating effects of behavioral regulation. Specifically, the indirect effects of physical exercise participation goals on exercise behavior were mediated by intrinsic regulation and amotivation. This suggests that individuals who pursue physical goals such as fitness and health are more likely to internalize exercise as meaningful and enjoyable, thereby increasing their engagement. In addition, both physical and psychological goals appear to influence exercise behavior through introjected regulation. This pattern indicates that exercise participation may be driven not only by autonomous motives but also by internal pressures, such as the desire to avoid guilt. Overall, these findings provide partial support for H9.

This study also demonstrated significant mediating effects of exercise-participation goals. Specifically, physical and psychological goals were found to mediate the effects of both positive and negative affect on exercise behavior. This suggests that affective states may influence exercise not directly, but by shaping individuals’ expectations and motivations regarding exercise outcomes. Individuals experiencing heightened positive or negative affect may develop stronger exercise-related goals, which in turn increase their likelihood of engaging in exercise. Similarly, physical goals were found to mediate the relationship between perceived stress and exercise behavior, suggesting that higher stress levels may lead individuals to place greater importance on the physical benefits of exercise as a means of self-regulation, which in turn increases their likelihood of engaging in exercise. Overall, these findings provide partial support for H10.

### Unexpected findings under pandemic conditions

Several findings diverged from expectations in this study. First, both negative affect and perceived stress were positively associated with exercise participation goals, contrary to prior research suggesting that such states typically undermine motivation. One possible explanation is that pandemic-related uncertainty and emotional strain heightened individuals’ awareness of the regulatory benefits of exercise, leading them to assign greater importance to exercise-related goals as a means of coping. In this context, exercise may have been framed less as a leisure activity and more as a tool for managing psychological distress.

Second, psychological exercise goals were associated with introjected regulation rather than intrinsic motivation. This pattern may reflect the conditions of the pandemic, where exercise was often pursued to alleviate negative feelings (e.g., stress, boredom) rather than for enjoyment, thereby fostering controlled forms of motivation more.

Finally, coping ability was positively associated with exercise behavior but not with participation goals, suggesting a more direct pathway from coping to action. Under constrained and disrupted routines, individuals with stronger coping capacity may have been more likely to engage in exercise as an immediate self-regulation strategy, without relying on explicit goal-setting processes.

Taken together, these findings suggest that under pandemic conditions, exercise motivation may have shifted toward a more regulation-oriented pattern, where emotional and stress-related factors play a central role in shaping both goals and behavior.

### Theoretical and practical implications

This study's theoretical contribution is to integrate the constructs of behavioral regulation and participation goals and to examine the direct and indirect effects of leisure-time exercise behavior during the pandemic. The study reveals that physical participation goals, which reflect the physical outcomes individuals seek through exercise, are associated with regulatory processes that capture the underlying reasons for engaging in exercise behavior.

The effect of the pandemic period on leisure-time exercise behavior was also studied by incorporating factors such as individuals' affective state, perceived stress related to quarantine circumstances, and coping ability. The antecedent roles of affect and stress received limited attention in the literature. Therefore, examining the relationships between these constructs and exercise behavior is valuable.

The mediating role of physical and psychological goals on affect and exercise relationships is novel and represents an avenue for further investigation. To the best of the author's knowledge, no prior studies examined the indirect effects of positive and negative affect on exercise behavior through their influence on exercise participation goals. On that account, this finding makes a meaningful contribution. Likewise, the indirect effect of stress on exercise behavior, through its influence on physical goals, is meaningful.

The findings of this study offer insights that may inform exercise promotion campaigns and intervention programs, particularly in contexts similar to the COVID-19 pandemic. For instance, psychological goals, such as reducing feelings of depression, and physical goals, such as increasing energy and improving health, could be emphasized in campaigns to encourage exercise participation. Moreover, since intrinsic and introjected regulation were associated with exercise behavior, messages highlighting the enjoyment of exercise, or the avoidance of guilt and shame, might be more persuasive in motivating individuals to engage in physical activity.

The study also suggests that increased confinement and negative affect are associated with reduced exercise behavior. While these findings are correlational and do not imply causation, they align with prior research indicating a vicious circle between exercise, affect, and stress [24]. For example, reduced exercise may exacerbate negative affect and feelings of confinement, creating a feedback loop. Although this study cannot confirm causality, it raises the possibility that interventions targeting individuals experiencing high levels of confinement and negative affect could help break this cycle. For instance, public health authorities might consider developing and promoting daily in-home physical activity regimens tailored to individuals facing such challenges. Furthermore, the study highlights the potential role of stress coping in promoting exercise behavior. The findings suggest that providing individuals with tools to manage stress, such as meditation, breathing techniques, or counseling [55], might support their physical activity levels, particularly during stressful periods like the pandemic.

Finally, while the study's findings are rooted in the unique context of the COVID-19 pandemic, they may offer preliminary insights for post-pandemic scenarios, particularly in situations where individuals face similar stressors, such as social isolation or heightened anxiety.

### Limitations and future research

The study has some limitations. First, a set of limitations relates to the characteristics of the sample. Using a student sample limits the generalizability of the findings. Future research with a representative sample might validate the findings and extend their applicability. Non-respondents may differ systematically in stress, motivation, or exercise behavior, which could further limit generalizability.

Second, the study relies on self-reported measures, which may be subject to biases such as social desirability or recall inaccuracies. Observational measures of exercise behavior might be used to replicate the current research. In addition, the confinement measure relied on a single self-reported item comparing current behavior to pre-pandemic routines, which may introduce some ambiguity for individuals with already low mobility levels prior to the pandemic.

The study's cross-sectional design limits the ability to establish causal relationships. The results should, therefore, be interpreted with caution, bearing in mind that the relationships are correlational and not causal. Experimental studies that control for the explored variables and longitudinal studies that measure changes in exercise behavior over different periods might help reveal the causal relationships.

Furthermore, the study's focus on the COVID-19 pandemic, while providing a unique context for examining exercise behavior, may limit the generalizability of the findings to non-pandemic settings. The pandemic introduced unprecedented challenges, including lockdowns, social isolation, and heightened stress, which may have influenced participants' exercise habits in ways that are not typical. Future research should investigate whether the observed relationships hold across different contexts, such as during post-pandemic recovery or other public health crises.

Finally, while the study incorporates stress, affect, and coping within the SDT framework, it does not fully capture the complexity of these constructs or their interactions. For example, the study focuses on general stress and affect rather than specific pandemic-related stressors (e.g., fear of infection, financial instability) or discrete emotions (e.g., anxiety, boredom). Furthermore, affect and stress-related measures might be subject to recall bias. Exercise behavior was assessed over the previous seven days to obtain more accurate reports of the frequency and intensity of different types of physical activity, as such details are more likely to be forgotten over longer recall periods. However, affect, stress, and confinement-related variables were measured over the past month. The objective was to capture participants’ broader emotional experiences during the pandemic rather than focusing on transient fluctuations. However, because participants might have relied more on general perceptions than on particular experiences, the longer recall interval might have made them more vulnerable to recall bias. Future research could explore these intricacies to provide a more comprehensive understanding of how stress and affect impact exercise behavior.

Despite these limitations, the study offers valuable insights into the factors influencing exercise behavior during challenging times. By addressing these limitations in future research, scholars can build on the current findings to develop more robust frameworks and effective interventions to promote physical activity.

## Conclusions

This study examined how affect, stress-related constructs, exercise participation goals, and behavioral regulation jointly shape exercise behavior during the COVID-19 pandemic within a self-determination theory framework. Using structural equation modeling, the findings demonstrate that emotional states and stress influence exercise primarily through motivational processes, highlighting the central role of goals and regulatory styles in sustaining physical activity.

The study contributes to the exercise literature by extending SDT-based models to incorporate affective and stress-related factors and by testing both direct and indirect pathways linking these constructs to behavior. By modeling participation goals and behavioral regulation as mediators, the research provides a more integrated account of how psychological states translate into exercise engagement.

Finally, by focusing on the pandemic context, this study addresses a critical gap in understanding exercise behavior under crisis conditions. The findings offer insight into how heightened stress and emotional reactions shape motivation during periods of disruption, with implications for supporting physical activity when routines and resources are constrained.

## Fundings

No fundings was received for the preparation of this manuscript.

## Supplementary Information

Supplementary Material 1.

## Data Availability

The data and materials are available from the corresponding author upon request.
