# Supplementary material for: Exercise unveiled: an integrated model of motives, goals, affective states, and stress during the COVID-19 pandemic
Source: BMC Psychol. 2026 May 9;14:953. doi: 10.1186/s40359-026-04693-1 (PMC13326566; doi:10.1186/s40359-026-04693-1)
Supplement: Supplementary file 1 — Supplementary Material 1. [file 40359_2026_4693_MOESM1_ESM.docx]

**Behavioral regulation in exercise (Markland & Tobin, 2004)**

We are interested in the reasons underlying peoples decisions to engage, or not engage in physical exercise. Using the scale below, please indicate to what extent each of the following items is true for you. (0 - Not true for me / 4- Very true for me)

- Because other people say I should
- Because I feel guilty when I don’t exercise
- Because I value the benefits of exercise
- Because I think exercise is fun
- I don’t see why I should have to exercise
- Because my friends/family/partner say I should
- Because I feel ashamed when I miss an exercise session
- Because it’s important to me to exercise regularly
- I can’t see why I should bother exercising
- Because I enjoy my exercise sessions
- To please other people
- I don’t see the point in exercising
- Because I feel like a failure when I haven’t exercised in a while
- Because I think it is important to make the effort to exercise regularly
- Because I find exercise a pleasurable activity
- Because I feel under pressure from my friends/family to exercise
- Because I get restless if I don’t exercise regularly
- Because I get pleasure and satisfaction from participating in exercise
- I think exercising is a waste of time

**Exercise Behavior (Godin & Shepard, 1985)**

In the last three months, in a typical 7-Day period (a week), how many times on the average do you do the following kinds of exercise for more than 15 minutes during your free time ?

- Strenuous Exercise (heart beats rapidly) (e.g., running, heavy lifting, football, basketball, aerobics, or fast bicycling)
- Moderate Exercise (not exhausting) (e.g. Fast walking, easy bicycling, dancing)
- Mild Exercise (minimal effort) (e.g. Yoga, easy walking)

During a typical 7-Day period (a week), in your leisure time, how often do you engage in any regular activity long enough to work up a sweat (heart beats rapidly)? (1 - Never/ 5- Very Often)

**Exercise-participation goals (Rogers et al., 2008)**

Some positive outcomes expected to be achieved by physical activity are listed below as items. Please indicate how often each item contributed to get you to engage in exercise during the last three months. (1 - Never/ 5- Very Often)

- Building muscle strength
- Increased physical fitness
- Increased energy
- Improving health
- Feeling less bored
- Feeling less stressed
- Less depression

**Affect (Riediger et al., 2011)**

How much did you experience each of the six feelings mentioned below during the last three months? (0 - Not at all / 6- Very much)

- Joyful
- Content
- Interested
- Angry
- Anxious
- Low-spirited

**Level of perceived stress (Littman et al., 2006)**

How would you rate your ability to handle stress? (1 - I can shake off stress/ 6- Stress eats away at me)

**Ability to cope with stress (Littman et al., 2006)**

During the last three months, how would you rate the amount of stress in your life? (1 - no stress/ 6- Extreme stress)

**Confinement**

During the pandemic period, how often did you leave the house compared to the normal period? (1 - I left home as often as before the pandemic / 7- I stayed at home all the time)
